# Characteristics of invasive alien plants in different urban areas: the case of Kunshan City, Jiangsu Province, China

**DOI:** 10.3389/fpls.2025.1539457

**Published:** 2025-03-21

**Authors:** Yubing Liu, Yueheng Ren, Hua Zhang, Dongdong Qiu, Yanpeng Zhu

**Affiliations:** ^1^ State Key Laboratory of Environmental Criteria and Risk Assessment, Chinese Research Academy of Environmental Sciences, Beijing, China; ^2^ State Environmental Protection Key Laboratory of Regional Eco-process and Function Assessment, Chinese Research Academy of Environmental Sciences, Beijing, China

**Keywords:** invasive alien plants, urban ecology, species diversity, phylogenetic diversity, sustainable development

## Abstract

As globalization progresses, the threat of invasive alien plants to ecosystems is becoming increasingly prominent, and the negative effects of these plants on human health and socioeconomics are gradually increasing with the development of cities; thus, concern about the problem of invasive alien plants in cities is gradually increasing. In this context, we analyzed the differences in the distribution characteristics of invasive alien plants in urban green space, countryside and farmland in Kunshan city, which is located in the Yangtze River Delta region, an area characterized by rapid urbanization. Additionally, the relations between local plant diversity and the intensity of human activities on invasive alien plants were explored. The following results were obtained: (1) There are 38 species of invasive plants in Kunshan, among which 9 species, such as *Alternanthera philoxeroides* and *Erigeron canadensis*, are distributed in all kinds of urban areas. There are no endemic invasive plants in the urban green space; however, *Amaranthus blitum* and eight other species are distributed only in the countryside, and seven species, such as *Bidens pilosa*, are found only in farmland areas. (2) In different urban areas, native plant species and phylogenetic diversity vary in their resistance to invasive alien plants. Compared with those in other areas, the coverage and importance values of alien invasive plants in the urban countryside significantly decreased with increasing quantity of native plant species and phylogenetic diversity. (3) GDP per capita, the proportion of built-up land and road density were the main factors affecting the distribution of invasive alien plants, but there were differences in the influence of human activities in different urban areas. The importance values of invasive alien plants increased significantly with increasing population density and GDP per capita in the countryside, but there was no such trend in urban green space or farmland areas. Overall, these findings suggest that urban planning and landscape management strategies should target the management of invasive alien plants based on the characteristics in different urban areas to maintain the stability and sustainability of urban ecosystems.

## 1 Introduction

With the acceleration of global urbanization, the structure and function of urban ecosystems are constantly changing (Wang et al., 2020; Holzmann et al., 2023; Román-Palacios and Wiens, 2020). Urban expansion and land use changes have led to the destruction or replacement of native natural vegetation, resulting in the fragmentation of urban habitats and declining biodiversity (Walsh et al., 2016), and artificially disrupted habitats can have different degrees of restriction on native species, whereas invasive alien plants can adapt in these areas (Liu et al., 2021); moreover, invasive alien plants are more capable of coping with environmental changes than are native plants, for example, some studies have shown that invasive alien species are better able to adapt to global climate change than native species are (Jia et al., 2016; Herben et al., 2004) and that invasive alien plants can quickly enter artificially disrupted habitats to compete with native species for survival space by utilizing their strong reproductive ability to adapt to these environments (Bradley et al., 2010; Valladares et al., 2015).

The proliferation of invasive alien plants in urban areas not only affects the survival of native plants but also profoundly affects the local ecological environment and human life (Potgieter et al., 2022; Seebens et al., 2020). Studies have shown that invasive plants have obvious preferences for different land uses in urban environments (Godefroid and Ricotta, 2018), their distribution in urban environments is spatially variable (Štajerová et al., 2017), and the ecological heterogeneity of urban landscapes can drive significant changes in the invasion process of invasive plants (Wang et al., 2019). Therefore, it is important to understand the distribution patterns of invasive plants in heterogeneous urban environments and explore the impact mechanisms of invasive plants in different urban areas, which can provide a basis for decision-making and the scientific management of biological invasions.

In 1958, Charles Elton, a British ecologist, proposed the “diversity-invasibility hypothesis”, which states that the higher the species diversity of native communities is, the stronger the resistance to the invasion of exotic plants and the lower the probability of successful invasion of exotic plants (Erickson and Elton, 1960). This may be because the greater the species diversity is, the more complex the interactions between species in the plant community and the greater their ability to resist invasion by exotic plants (Beaury et al., 2020). Additionally, the abundance, variety, and composition of native species can affect invasion by exotic plants (Levine, 2000; Dukes, 2001; Levine et al., 2004; Pearson et al., 2018). In addition to species diversity, some studies have suggested that the phylogenetic diversity of plants in native communities is closely related to the successful invasion of exotic plants (Li et al., 2015; Feng and Kleunen, 2016; Malecore et al., 2019; Fridley et al., 2007). One study showed that phylogenetic diversity buffers the extent of invasion by exotic trees (Delavaux et al., 2023). When native communities have high phylogenetic diversity, their species tend to display well-established patterns and strong adaptations in terms of resource utilization, ecological niche occupancy, and ecological functions (Kraft et al., 2011); this complexity and adaptability may enable native communities to compete more effectively with invasive alien plants for resources and thus inhibit the invasion and spread of alien species (Catford et al., 2009).

The results of several studies of urban invasive alien plants globally indicate that urbanization has had a significant effect on the species, distribution and invasion mechanism of invasive alien plants (Yang et al., 2009). For example, land use changes during urbanization (e.g., the construction of urban green spaces and landscape gardens) may unintentionally introduce exotic plants. Moreover, high-use transportation and logistics networks in cities, as well as night-time light pollution caused by the addition of streetlights during urbanization, facilitate the spread of exotic plants and promote the invasion of some exotic plants (Speißer et al., 2021; Liu et al., 2022; Murphy et al., 2022). Warmer climates, an abundant nutrient supply and few natural enemies in urban artificial environments are also conducive to the growth and reproduction of invasive alien plants (Li et al., 2005).

There have been few studies of the distribution patterns and invasion mechanisms of alien invasive plants in different urban areas. Therefore, in this study, Kunshan city, which is located in the Yangtze River Delta region and has experienced rapid urbanization and economic development, is selected as the research area, and the alien invasive plants in Kunshan city are selected as the research objects. On the basis of systematic vegetation surveys, we analyzed the composition and distribution characteristics of alien invasive plants in different urban areas and explored the relationships between the species diversity and phylogenetic diversity of native plants and alien invasive plants. Moreover, we identified the important factors affecting the invasion of invasive plants, with the aim of providing a scientific basis and suggestions for ecological protection, urban planning and biodiversity maintenance.

## 2 Materials and methods

### 2.1 Study area

Kunshan (120°48′21″-121°09′04″E, 31°06′34″-31°32′36″N) is located in the Taihu Lake Plain of the Yangtze River Delta. The total area of the city is 931 km^2^, of which the water area accounts for 23.1%. The terrain is flat, with an overall hilly plain landscape. Since the 1990s, economic and social development has rapidly occurred in Kunshan, and by 2020, the urban area expanded by six times that in 1990, with the built-up area accounting for approximately 45% of the total area. Between 2010 and 2020, the resident population increased by 27.21%, and the GDP doubled. Via data reviews and onsite surveys, the number of invasive alien plant species was determined, and many of these species were found to be very harmful to the local environment.

### 2.2 Sample survey and data collection

**Figure 1:**
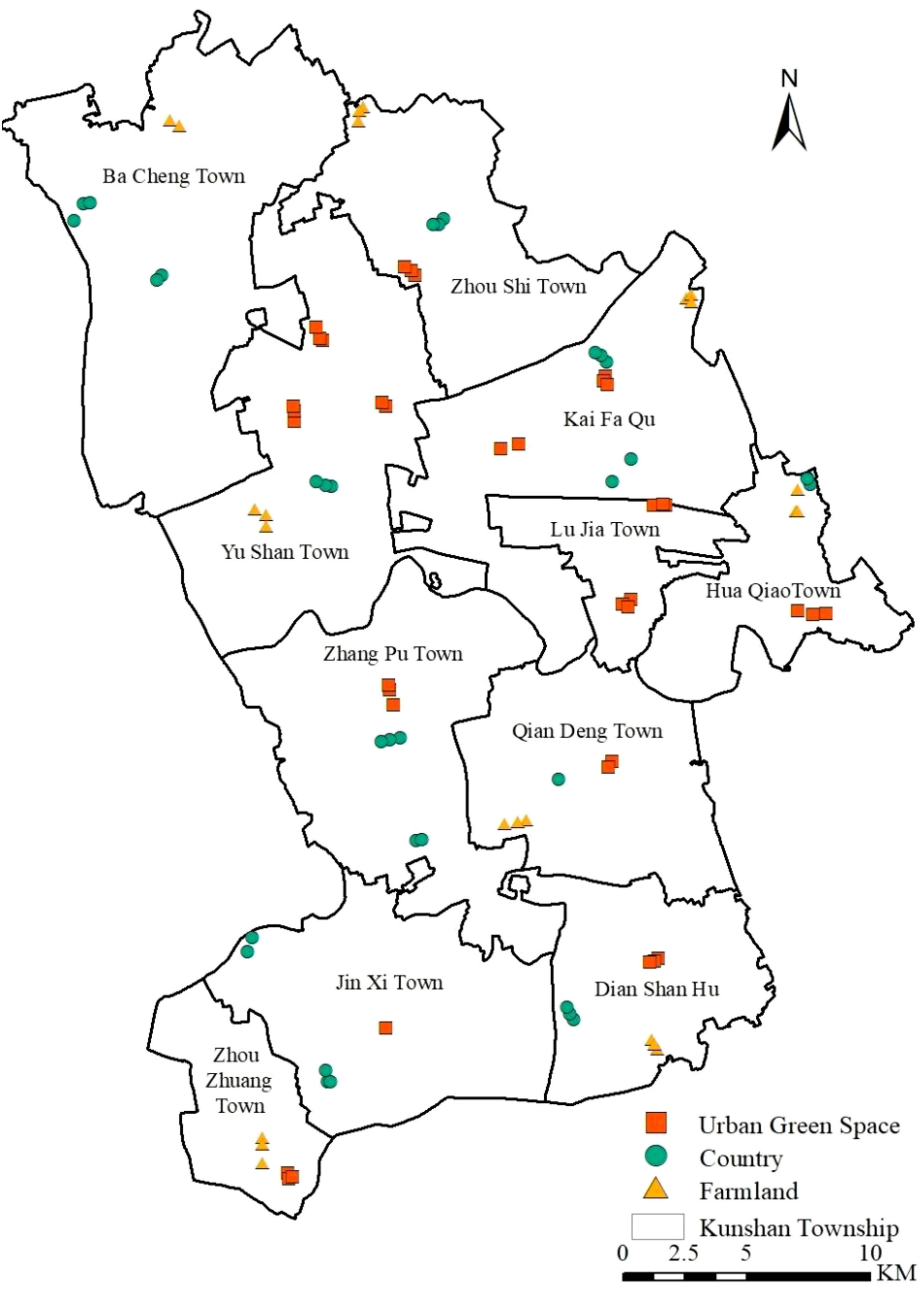
Sample Distribution of Invasive Alien Plants in Kunshan City.

The invasive alien plant survey points were selected considering various factors, such as the urban ecosystem, degree and type of anthropogenic interference, land use type, and level of road traffic. Finally, we selected three areas with different degrees of urbanization, namely, urban green space, countryside and farmland, for the survey. In July 2022, we systematically surveyed each sample plot via a combination of sample surveys and visual evaluation methods. At each sampling point, we set up a 10 m × 10 m tree sample, and four 1 m × 1 m herbaceous samples were obtained near the top of each tree sample. We recorded the name, number of trees, cover and height of the plant species in each layer of the tree–shrub–grass systems at each sample location at the time of the survey, and each plant was labelled to indicate if it was cultivated or not cultivated and whether it was an invasive alien species or not. Invasive alien plants were identified according to the China Invasive Alien Species Information System (https://www.iplant.cn/ias/) and the Chinese List of Invasive and Naturalized Plants 2023 Edition (https://www.cvh.ac.cn/iapc/). After comparison and labelling, we identified 93 sampling sites with invasive alien plants that were randomly distributed in different urban areas of Kunshan (**Figure 1**).

### 2.3 Data acquisition and analysis

#### 2.3.1 Selection of indicators

According to different invasion mechanisms, we selected invasive alien plant richness (S_I_), invasive alien plant coverage (IC), and invasive alien plant abundance importance value (IIV) as the indices to assess invasive alien plants. S_I_ was expressed as the number of species of invasive alien plants in the sample, and IC was expressed as the total coverage of all invasive alien plants in the sample.

We selected native plant species richness (S_N_), native plant species Shannon diversity (H_N_), and native plant species phylogenetic diversity (PD) as native plant indicators. The population density (POPD), GDP per capita (GDPP), built-up land area share (BUI), and road network share (ROA) were selected as urbanization environment indicators. The relationships between each indicator and the indices at the sampling sites were analyzed.

The source of population data and GDPP was the Kunshan 2020 Statistical Yearbook. The source of land use data was the 2020 land use vector data provided by the Resource Environment Science and Data Center (https://www.resdc.cn/) of the Institute of Geographic Sciences and Resources, Chinese Academy of Sciences, with an accuracy of 30 meters. The source of the road data was Open Street Map (OSM). A circle with a radius of 500 m with a sampling point as the center was created, and the density of the road network and the percentage of built-up land area in the circular area were calculated.

#### 2.3.2 Methods of data processing

The importance value indicates the relative importance of a plant species in a community, and IIV of each sample site was calculated as follows: importance value = (relative abundance + relative cover + relative frequency)/3.

We used the Keping Ma method at each sample site to calculate the abundance of native plants (S_N_) and the Shannon−Wiener index of diversity (H_N_) of native plants (Ma and Liu, 1994). The spectral diversity (PD) of the native plants was calculated at each sample site (Faith, 1992).

The construction land share was calculated via the following formula:


$$\;BUI=\frac{\sum_{i=1}^{S}s_{i}}{S},$$

where *BUI* is the road density index in the study area; *s_i_* is the area of different construction sites in terms of land use in square kilometers (km^2^); and *S* is the total area of the study area in square kilometers (km²).

The road density index was calculated via the following formula:


$$ROA=\frac{\sum_{i=1}^{5}l_{i}}{S},$$

where *ROA* is the road density index in the study area; *l_i_* is the length of different grades of roads in kilometers (km); and *S* is the total area of the study area in square kilometers (km²).

One-way ANOVA and Duncan tests were used to examine the differences in the richness, cover and importance values of invasive alien plants in different urban areas. Redundancy analysis (RDA) was used to explore the relationships between native plant diversity and the importance values of alien invasive plants, and a Monte Carlo replacement test of the analysis results was performed. Linear fitting analysis was used to explore the relationships between native plant species diversity and phylogenetic diversity and invasive alien plant abundance, cover, and importance values in different urban areas. Redundancy analysis (RDA) was used to explore the relationships between environmental factors and the importance values of alien invasive plants, and a Monte Carlo replacement test of the analysis results was performed. Linear regression analysis was subsequently used to explore the relationships between environmental factors and in the index values of alien invasive plants in different urban areas.

The data indices were calculated and analyzed in R, the Venn Diagram package was used for compositional analysis of invasive alien plants, the vegan package for species diversity calculations and RDA analyses, the picnate package for phylogenetic diversity calculations, and the ggplot2, ggpubr packages for mapping analyses.

## 3 Results

### 3.1 Composition of invasive alien plants in Kunshan

We recorded a total of 302 plant species in our surveys, belonging to 91 families and 236 genera, among which 38 species of invasive plants spanned 13 families and 30 genera. Among the sample points, those containing invasive plants accounted for 87.74% of all sample points, indicating that the invasive plant problem in Kunshan is serious. There were 18 species of invasive alien plants in urban green areas, 30 species of invasive alien plants in the countryside, and 22 species of invasive alien plants in farmland areas. In terms of invasive grade, according to the national invasive grade classification of the “China Invasive Alien Plant List” (Ma and Li, 2018), there are 7 species with the invasive grade 1 in urban green areas, 8 species of invasive grade 1 in the countryside, and 9 species of invasive grade 1 in farmland areas. In terms of origin, most invasive alien plants in urban green spaces, countryside areas and farmlands originated from America, with 16, 22 and 16 species, respectively. In terms of life forms, there were 10 species of invasive annuals in urban green spaces, 17 species of invasive annuals in the countryside, and 18 species of invasive annuals in farmland areas.

**Figure 2:**
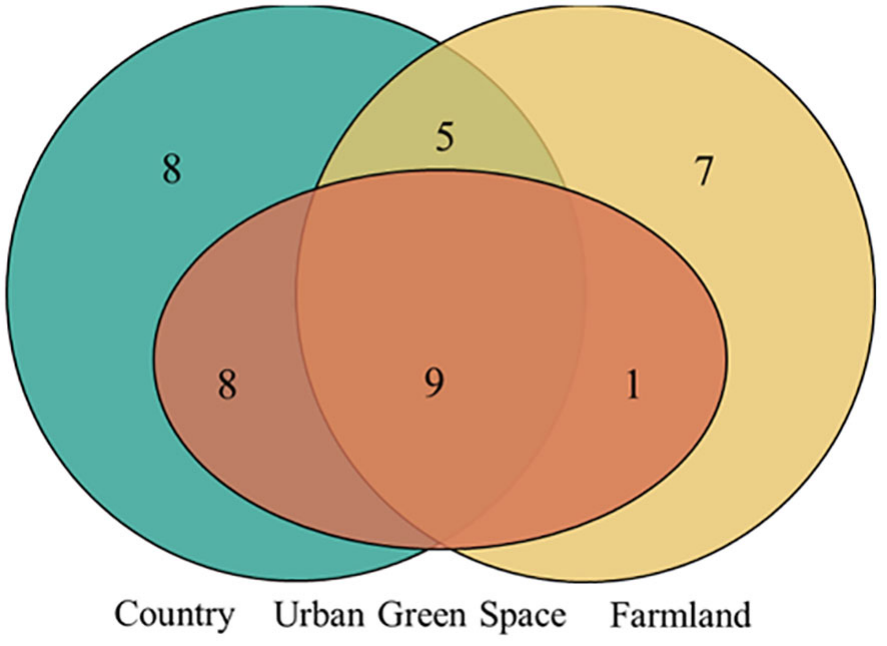
Venn diagram of the distribution of invasive alien plants in different urban areas. Orange represents urban greenspace, green represents countryside, and yellow represents farmland. The overlap of the three represents the distribution of 9 invasive plants in the three urban areas; the overlap of orange and green represents the distribution of 8 invasive plants in urban greenspace and countryside, the overlap of orange and yellow represents the distribution of 1 invasive plant in urban greenspace and farmland, the overlap of green and yellow represents the distribution of 5 invasive plants in countryside and farmland; only green represents the distribution of 8 invasive plants only in the countryside, and only yellow represents the distribution of 7 invasive plants only in the farmland.

Among all the invasive alien plants surveyed (**Figure 2**), nine species were distributed in urban green spaces, countryside areas and farmland areas, namely, *Euphorbia maculata*, *Bidens frondosa*, *Amaranthus retroflexus*, *Solidago canadensis*, *Sonchus oleraceus*, *Alternanthera philoxeroides*, *Erigeron canadensis*, *Erigeron annuus*, and *Symphyotrichum subulatum*. Eight invasive alien plants were distributed only in the countryside, namely, *Amaranthus blitum*, *Amaranthus tricolour*, *Melilotus officinalis*, *Cosmos bipinnatus*, *Oxalis corymbosa*, *Coreopsis basalis*, *Amorpha fruticose*, and *Gaura lindheimeri*. Seven invasive alien plants were distributed only in farmland areas, namely, *Bidens pilosa*, *Ricinus communis*, *Abutilon theophrasti*, *Oxybasis glauca*, *Physalis angulate*, *Sonchus asper*, and *Gaillardia pulchella*.

### 3.2 Characteristics of invasive alien plants in different urban areas

**Figure 3:**
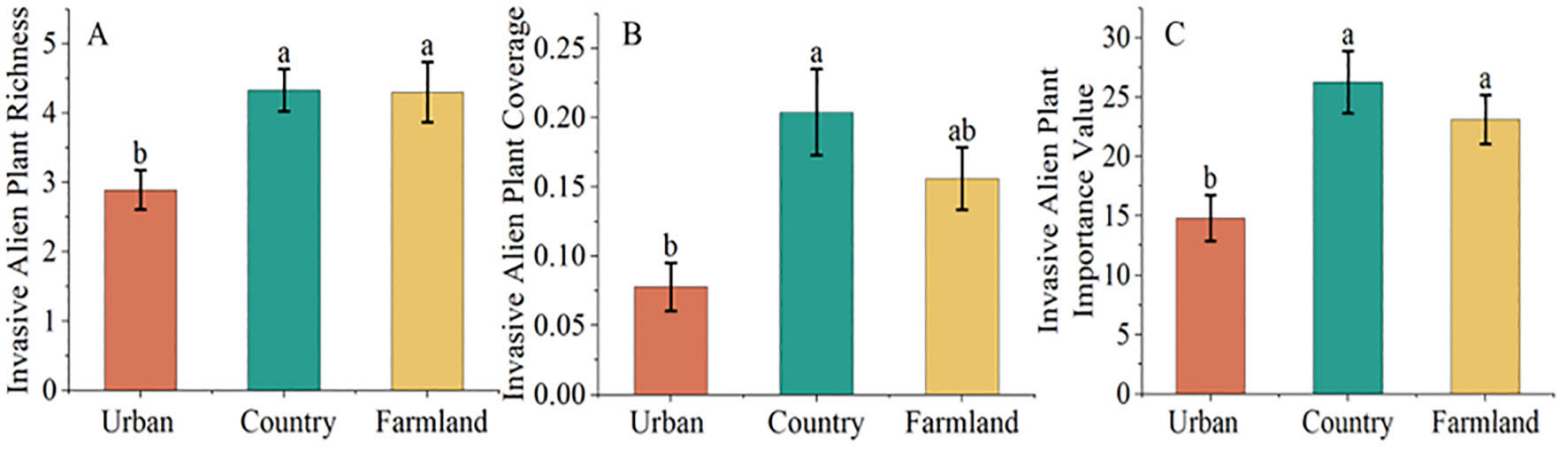
Differences in invasive alien plant richness **(A)**, coverage **(B)**, and importance value **(C)** across different urban areas. The data are the means ± SEs. Different lowercase letters indicate significant differences according to Duncan’s test (P<0.05).

The results of one-way ANOVA (**Figure 3**) revealed that alien invasive plant richness (F=6.791, P=0.002), alien invasive plant cover (F=7.584, P<0.001), and the alien invasive plant importance value (F=7.606, P<001) differed significantly. The results of multiple comparisons indicated that alien invasive plant richness, cover, and importance value were significantly greater in the countryside than in the urban green space. The abundance and importance value of invasive alien plants in farmland areas were significantly greater than those in urban green spaces, and the abundance, cover and importance value of invasive alien plants in the countryside and farmland areas were not significantly different.

### 3.3 Impact of native plant diversity on invasive alien plants in different urban areas

**Figure 4:**
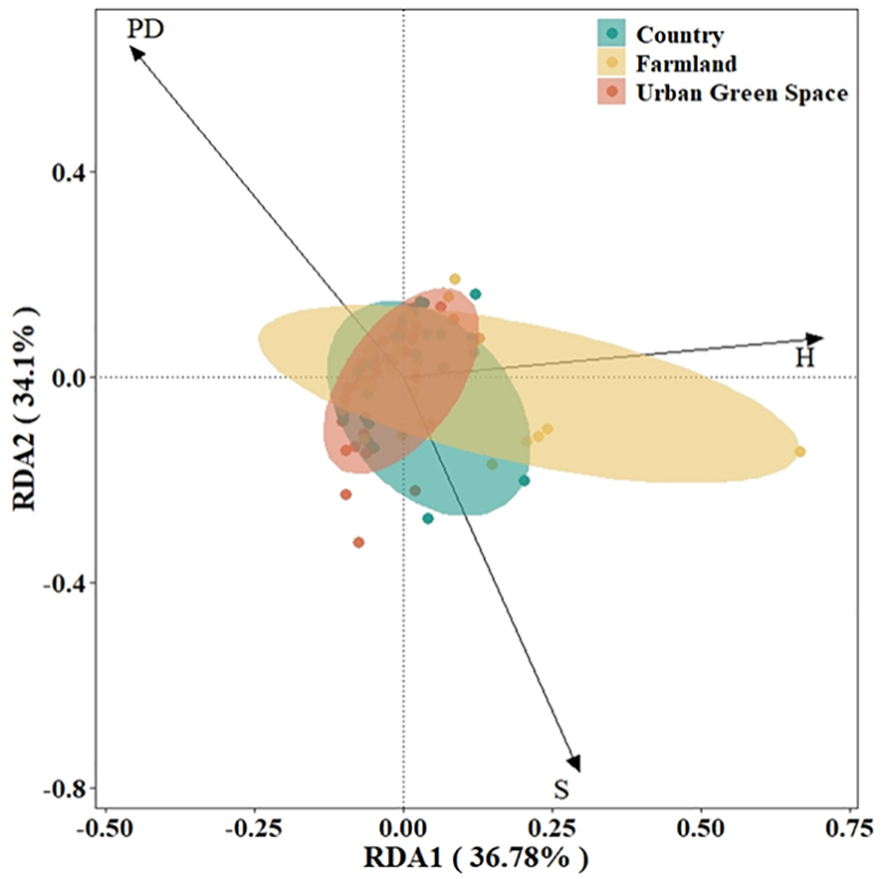
RDA rankings of importance values of invasive alien plant and native plant diversity in Kunshan city, China. S for native plant richness, H for native plant Shannon diversity, PD for native plant phylogenetic diversity.

On the basis of the DCA results (axis length<4.0), RDA was selected to rank and analyze the exotic invasive plant importance values and native plant diversity data in different urban areas. The results of the RDA showed that S_N_ and PD contributed mainly to RDA axis 1 and that H contributed mainly to RDA axis 2 (**Figure 4**), with the first principal component explaining 36.78% of the variation and the second principal component explaining 34.1%; the two axes together encompassed the contribution of 70.88% of all the variables. As shown in the figure, the results of the RDA indicate a positive correlation between S for countryside invasive alien plant importance values and H for farmland invasive alien plant importance values.

The results of the Monte Carlo replacement test revealed that S, H, and PD are all native plant-related factors that influence the importance values of invasive alien plants (**Table 1**).

**Table 1:** Importance values of invasive alien plants and the results of native plant diversity replacement tests in Kunshan.

	R^2^	P	
S	0.1445	0.001	***
H	0.2043	0.001	***
PD	0.1601	0.002	**

*** means P<0.001, there is a highly statistically significant difference; ** means P<0.01, statistically significant difference.

**Figure 5:**
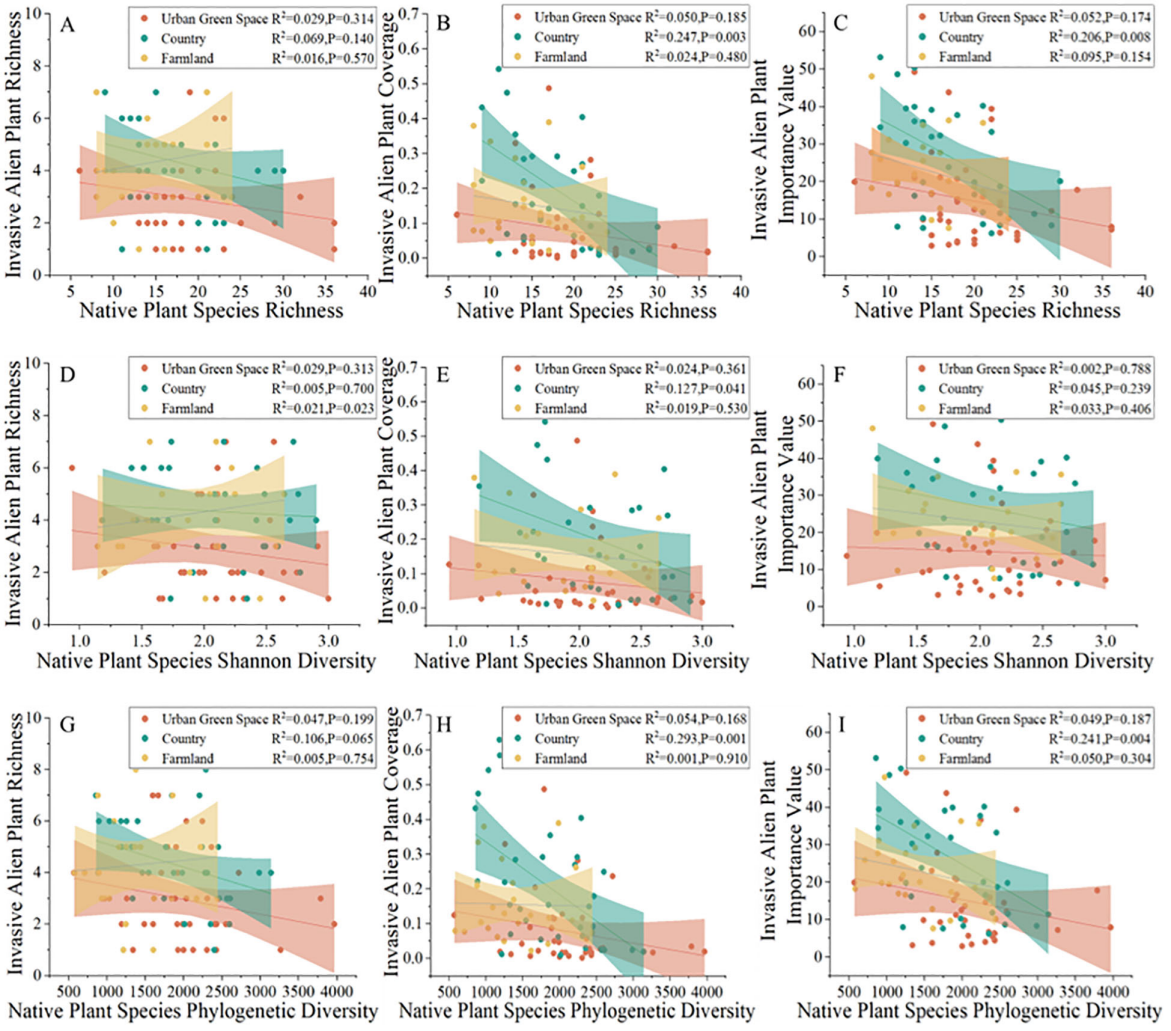
Relations between the native plant richness **(A-C)**, Shannon’s diversity index **(D-F)**, phylogenetic diversity index **(G-I)** and invasive alien plant richness, coverage and importance values in different urban areas.

The analysis of the results revealed (**Figure 5**) that in the countryside, the cover of invasive alien plants (R^2^ = 0.247, P=0.003) and the importance values of invasive alien plants (R^2^ = 0.206, P=0.008) significantly decreased with increasing richness of native plants. There was no significant trend in the Shannon diversity index of native plants or invasive alien plants. As the diversity index of the native plant spectrum increased, the cover of invasive alien plants (R^2^ = 0.293, P=0.001) and the importance values of invasive alien plants (R^2^ = 0.241, P=0.004) significantly decreased. Areas of native plants with high phylogenetic diversity (PD) display a negative relationship with the abundance of exotic invasive plants. The relations between native plant richness, the Shannon’s diversity index of native plants, and phylogenetic diversity and invasive alien plants were all significant in urban green spaces and farmland areas.

### 3.4 Environmental impacts of invasive alien plants in different urban areas

**Figure 6:**
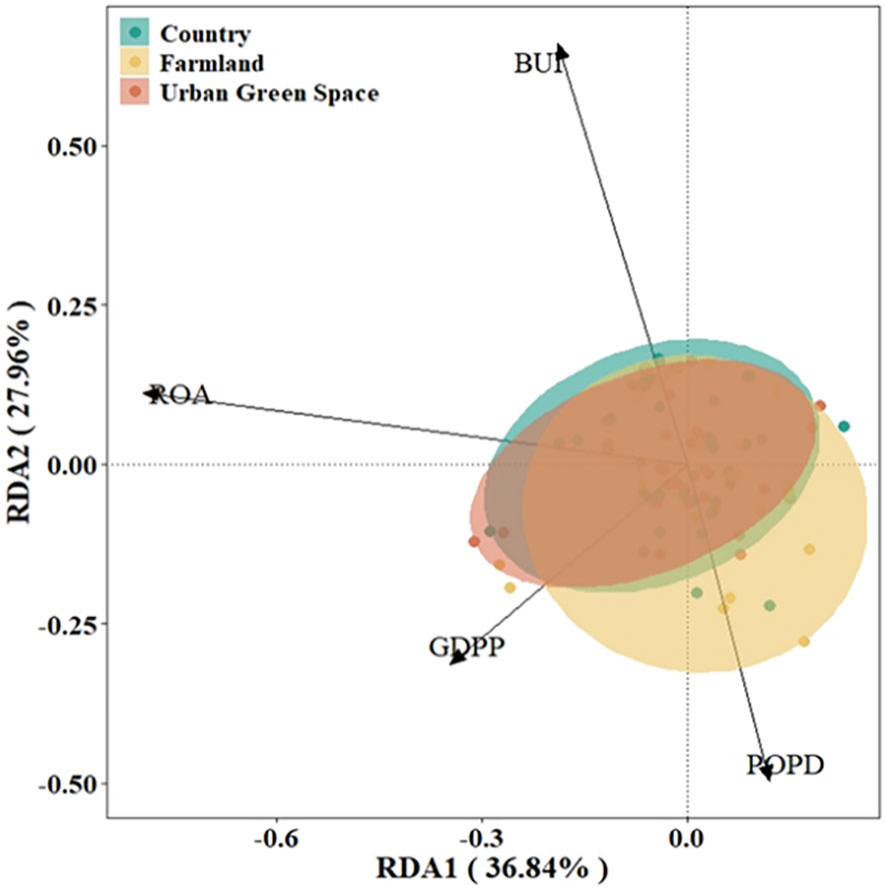
RDA rankings of the importance values of invasive alien plants on the basis of environmental factor data in Kunshan city, China. POPD for population density, GDPP for per capita GDP, BUI for built-up land area share, and ROA for road network share.

On the basis of the DCA results (axis length<4.0), RDA was selected to rank and analyze the importance values of alien invasive plants in different urban areas. The confidence ellipses for urban green space and the countryside were relatively small, indicating high consistency among the samples, whereas the confidence ellipse for agricultural land was larger, indicating greater internal variability. The results of the RDA showed that ROA and GDPP mainly contribute to RDA axis 1 and that BUI and POPD mainly contribute to RDA axis 2 (**Figure 6**). The first principal component explains 36.84% of the variation, and the second principal component explains 27.96%; the two axes together encompass the contribution of 64.80% of all the variables. The results of the RDA indicate the strong influence of the environmental factor GDPP on the importance values of green spaces and the countryside in urban areas.

The results of the Monte Carlo replacement test revealed that the GDPP, BUI and ROA are variables correlated with the distribution of alien invasive plants (**Table 2**), and the importance values of alien invasive plants in Kunshan city are highly significantly correlated with the BUI and ROA (P<0.01) and significantly correlated with the GDPP (P<0.05).

**Table 2:** Importance values of invasive alien plants and the environmental factor replacement test results in Kunshan.

	R^2^	P	
POPD	0.0716	0.057	
GDPP	0.0808	0.027	*
BUI	0.1293	0.009	**
ROA	0.2418	0.001	***

*** means P<0.001, there is a highly statistically significant difference; ** means P<0.01, statistically significant difference; * means P<0.05, fhere is a difference.

**Figure 7:**
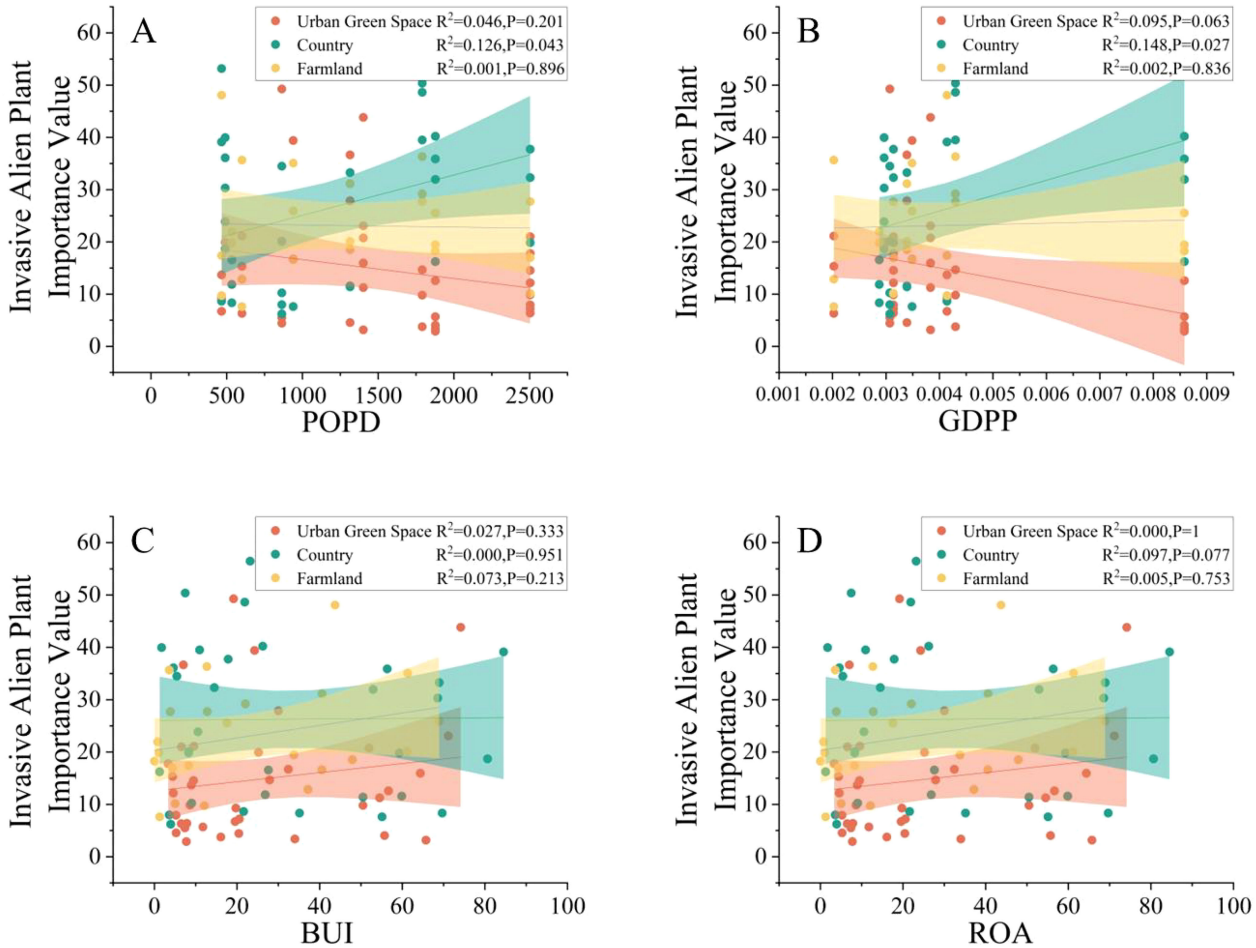
Relations between POPD **(A)**, GDPP **(B)**, BUI **(C)**,ROA **(D)** and invasive alien plant importance values in different urban areas.

A linear regression analysis of the importance values of alien invasive plants in different urban areas with respect to environmental factors was conducted, and the results are shown in **Figure 7**; notably, in the countryside, the importance values of alien invasive plants (R^2^ = 0.126, P=0.043; R^2^ = 0.148, P=0.027) significantly increased with increasing POPD and GDPP, which indicated that in the countryside, POPD and GDPP are the main factors related to alien plant invasion.

## 4 Discussion

### 4.1 Characteristics of invasive alien plants in different urban areas

Among the 38 species of invasive alien plants investigated in this study, 18 species were in urban green spaces, 30 species were in the countryside, and 22 species were in farmland areas. In terms of origin, the most invasive alien plants in urban green spaces, countryside areas and farmland areas originated from America, with 16, 22 and 16 species, respectively; the results of this study were the same as those of studies on the status of invasive alien plants in Zhanjiang (Duan et al., 2022). In terms of life type, annual herbaceous plants accounted for the largest proportion of sampled invasive species; these plants are characterized by a short growth cycle and high adaptability (Xi et al., 2021), with 10, 17, and 18 species, respectively. In different urban areas of Kunshan, there were 9, 13, and 11 species of Asteraceae, as previously indicated in a survey of alien invasive plants in the built-up areas of Shenzhen (Li et al., 2024), with Asteraceae being the most abundant alien invasive plant (Qiang and Ma, 2022). Among all the investigated alien invasive plants, nine species were distributed in urban green areas, the countryside and farmland areas, and invasive class I species accounted for the largest proportion of the nine species, indicating that the invasive ability of alien plants is widely distributed.

The distribution of invasive alien plants in different urban areas is characterized by species differentiation and a structure with notable spatial variability. In this study, the abundance, cover, and importance values of invasive alien plants were lowest in the urban green space; the abundance, cover, and importance values of invasive alien plants in the countryside were significantly greater than those in the urban green space; and the abundance and importance values of invasive alien plants in the farmland were significantly greater than those in the urban green space. As previously noted in an invasive alien plant study in Beijing, the diversity of invasive alien herbaceous plants increased as the urbanization gradient decreased, and the richness of invasive alien plants in parks in the urban core area was lower than that in adjacent areas and the suburbs because environmental factors are the main factors affecting the distribution of invasive plants in remote areas of the city, whereas human activities are the main factors affecting the distribution of invasive plants in highly urbanized areas (Zhang, 2014; Zhao et al., 2022). The results are influenced by the significant differences in the ecological conditions and human activity patterns in different urban areas. In urban green spaces, the influence of environmental factors is lower, and the influence of human activities is greater. Plants in urban green spaces are usually artificially maintained and managed on a regular basis to prevent the growth of invasive plants, protect native vegetation and maintain an ecological balance; in turn, the vegetation species and distribution in green spaces are relatively stable. For example, in one study, management priority criteria were developed for invasive plants in the city of Cape Town to ensure the stabilization of the urban environment (Potgieter et al., 2018). Management practices and the management intensity have a greater impact on invasive alien plants than do other factors. Invasive alien plants that are able to stably survive in urban green spaces have the ability to adapt to the environment of the urban green space, and only those that are adapted to high levels of disturbance and are able to survive with limited resources are able to survive for a long period in urban green spaces (Munné-Bosch and Santos, 2024). In the countryside, the influence of environmental factors is greater than that in other areas, and the influence of human activities is smaller. Due to the lower level of human interference, the environment is more natural and the ecosystem is generally well established; therefore, it is easy for exotic plants to grow and spread in these areas. The ecological environment in the countryside is comparatively complex and includes diverse habitats and ecological niches in the community; therefore, it can accommodate different types of invasive alien plants. A study of the distribution of invasive plants in the urban and rural areas of Cape Town showed that the countryside can accommodate both urban invasive alien plants introduced as horticultural ornamental plants and alien invasive plants from farmland areas (Afonso et al., 2020).

### 4.2 The resistance of native plants in areas with different diversity levels to invasive alien plants varies across urban areas

Resistance to invasive alien plants varies with native plant diversity in different urban areas, with resistance to invasive alien plants gradually increasing with increasing native plant diversity in relatively more natural countryside areas, but this trend does not occur in urban green spaces or agricultural areas. The diversity-invasiveness hypothesis suggests that the greater the diversity of native plants is, the greater the resistance to alien invasions (Erickson and Elton, 1960). In urban green spaces and agricultural areas, the results of this study are inconsistent with the diversity-invasiveness hypothesis. In the countryside, the results of this study are consistent with the diversity-invasiveness hypothesis. The results of the present study are similar to those of a study based on data from native plant communities, in which species and phylogenetic diversity differed at different stages of invasion in response to alien plant invasions, suggesting that the biotic resistance hypothesis may be stage dependent, a result that is also supported by the findings of the present study (Guo et al., 2024).

The cover and importance values of alien invasive plants were significantly negatively correlated with native plant abundance in the countryside, and there was no correlation in urban green spaces or farmland areas. The results of this study were the same as those of other studies of resistance to invasive species in different habitats in tropical forest areas. The correlation between native species richness and alien invasive plant indicators varies in different habitat environments (Mungi et al., 2021) because spatial heterogeneity in cities influences the correlation between native plant species diversity and exotic invasive plants (Wang et al., 2019), high resource availability makes these areas ideal for invasive species, allowing them to establish in different communities (Karrer et al., 2020), and exotic invasive plants have different preferences for different land types in urban environments (Godefroid and Ricotta, 2018). Recent studies have shown that high biological species diversity is associated with increased resistance of grasslands to plant invasions under multiple environmental changes (Cai et al., 2024), and it is hypothesized on the basis of the results of this study that resistance to invasive alien plants may increase in relatively natural areas as native plant species diversity increases. In the countryside, where land use types are relatively diverse, there may be a variety of habitats interspersed and low levels of anthropogenic interference, the ecological environment may be more complex, the competition between species may be intense, and herbaceous plant communities with high species diversity have high biotic resistance to invasive plants because most of the ecological niches are occupied by native species, resulting in limited resources available to invasive alien plants (Yu and Li, 2020). In contrast, there are more anthropogenic influences in urban green spaces and agricultural areas, which cannot be ignored when conducting diversity-invasiveness analyses.

The cover and importance values of invasive alien plants in the countryside were significantly negatively correlated with the PD of native plants. The larger the community PD value is, the more ecological niches the community occupies and the more stable the community is. High phylogenetic diversity of native plants is associated with reduced invasion ability for alien invasive plants (Ernst et al., 2022). Habitats in the countryside are comparatively more intact than those in other areas, the phylogenetic diversity of native plants is usually greater, and plant communities are able to form complex ecological networks with strong stability and adaptability through long-term natural selection and evolution. Thus, resistance to exotic invasive plants is strong.

In summary, the resistance of native plants in different urban areas to invasive alien plants varies, especially considering genetic diversity. In the countryside, with a relatively natural environment, the diversity-invasiveness hypothesis is verified. Therefore, in urban planning and ecological conservation, the protection and enhancement of native plant diversity should be emphasized according to field conditions to increase resistance to invasive alien plants.

### 4.3 The environmental factors affecting invasive alien plants vary in different urban areas

The results of the RDA showed that GDPP, BUI and ROA are the environmental factors most highly correlated with invasive alien plant abundance in Kunshan and the importance values of invasive alien plants, indicating that invasive alien plants are affected by integrated environmental factors. Additionally, the contribution of POPD is small compared with that of other environmental factors. The reasons for this may be the high urbanization of the study area, the higher the GDPP the greater the BUI and ROA, the high degree of fragmentation of the urban landscape due to the construction of built-up land, which provides suitable habitat for invasive alien plants, and the density of the roads where the spread of invasive alien plants can be carried out by vehicles. In the city the daily range of human activities is limited therefore the impact is less.

The environmental factors affecting invasive alien plants vary in different urban areas compared to the environmental factors affecting invasive alien plants in Kunshan. The invasion of alien plants in the countryside increases with the population density and GDP per capita, where there is no such trend in urban green space or farmland. The reason for this may be that in urban areas, people are more focused on their work and life, and they will not take active measures to intervene to limit the growth of invasive alien plants in urban green space and farmland, which will not have a direct impact on invasive alien plants, and only a few sanitation personnel or park managers will carry out the corresponding management. A high GDP per capita does not mean that a large amount of funds will be invested in the prevention and control of invasive alien plants in urban green spaces and farmland areas. Moreover, in urban fund allocation, economic development, infrastructure development, and social welfare are often prioritized. Unlike urban green space and farmland, in the countryside, in the countryside, a high population density means that more people many people are engaged in local activities, such as excursions, camping, and hiking. These activities increase the contact between human beings and the natural environment, and people may unintentionally bring seeds of invasive plants into the countryside or destroy the local ecological environment to create suitable conditions for invasive plants to grow. Tourism and recreational activities are more common in areas with high per capita GDP in the countryside, and the development of countryside tourism may lead to more opportunities for the introduction of exotic plants.

In this study, alien invasive plants in Kunshan were highly significantly associated with built-up land and road density, as noted in a previous study on the probability of alien invasive plant occurrence in different land use types, which was related to the percentage of impervious surfaces (Deparis et al., 2022). However, the distributions of alien invasive plants in the three different urban areas of urban green space, countryside and farmland areas in this study were not correlated with the density of built-up land or roads; additionally, some studies have shown that the probability of occurrence of invasive plants is negatively correlated with the percentage of impervious surfaces in some habitats with strong anthropogenic activities, while a positive correlation was observed for habitats with relatively limited anthropogenic activities (Li et al., 2023). Other studies have suggested that the probability of occurrence of the alien invasive plant Rudbeckia laciniata increases with increasing road density (Munemitsu et al., 2015). However, the results of this study are different. The distribution of invasive alien plants in Kunshan is related to the density of construction land and roads, but there is no correlation between the different urban areas. The reason for this may be that invasive alien plant species and their distributions in different urban areas are different, and there may be differences in the distributions of invasive alien plants when analyzed together. For example, a study of the climatic ecological niche of invasive alien plants revealed that this niche is different from that of native alien species and that the climate in the native areas of otherwise invasive alien plants is warmer, drier, and more isothermal (Chen et al., 2024); therefore, invasive alien plants exist and survive in different habitats in different urban areas at small scales. Similarly, some invasive plants prefer to inhabit places with high densities of construction land and roads, whereas some invasive plants are negatively correlated with the density of roads in construction areas; thus, in analyses of invasive alien plants, the preferences of invasive alien plants as well as other comprehensive influencing factors should be considered.

### 4.4 Recommendations for the prevention and control of invasive alien plants in different urban areas

The species of invasive alien plants differ in different urban areas and display a strong spatial structure. The resistance of native plant diversity to invasive alien plants varies in different urban areas, and the environmental factors affecting invasive alien plants are different in different urban areas. On the basis of the results of this study of the invasive characteristics of alien plants in different urban areas, the following recommendations are made.

In urban green areas, the daily maintenance and management of green spaces should be strengthened to maintain a good ecological environment and reduce the living space for invasive alien plants. In the countryside, the relevant departments should strengthen the ecological protection of local areas and preserve the integrity of natural vegetation and ecosystems, such as by setting up nature reserves and expanding ecological corridors, to improve resistance to invasive alien plants. In areas where activities in the countryside are carried out, public awareness of invasive plant issues should be increased, with a focus on preserving the integrity of native plants, protecting natural ecosystems and increasing knowledge regarding the prevention of alien plant invasion. The common species of invasive alien plants in farmland areas and the associated risks should be publicized to increase awareness among residents so that they can detect and remove invasive alien plants in a timely manner during the cultivation of crops. Reasonable agricultural cultivation measures, such as crop rotation, intercropping and mulching, should be implemented to improve the stability of farmland ecosystems and reduce the chances of invasive alien plants surviving.

## 5 Conclusion

This study took Kunshan City, a city with rapid urbanization, as the study area, and analyzed the differences in the distribution characteristics of invasive alien plants in urban green space, countryside and farmland, and explored the mechanisms by which local plant diversity and the intensity of human activities affect invasive alien plants. The distribution of invasive alien plants in different urban areas showed species differentiation and strong spatial structure. 38 species of invasive alien plants found in Kunshan City, 9 species of invasive alien plants were distributed in all three urban areas, there were no endemic invasive alien plants in the urban green areas, 8 species of invasive alien plants were distributed in the countryside only, and 7 species of invasive alien plants were distributed only in the farmland.

The resistance of native plant diversity to invasive alien plants varied across urban areas, with alien invasive plant cover and importance values significantly decreasing with increasing native plant species diversity and phylogenetic diversity in the countryside compared to other urban areas. For the whole study area, invasive alien plants were influenced by a combination of environmental factors, and GDP per capita, proportion of built-up land and road density were the main factors influencing the distribution of invasive alien plants in Kunshan City. The environmental factors affecting the distribution of invasive alien plants were different in different urban areas. In the countryside, the importance of invasive alien plants increased significantly with the increase of population density and GDP per capita, but there was no such tendency in the urban green space and farmland.

Targeted management of invasive alien plants is recommended in urban planning and landscape management based on the characteristics of invasive plants in different urban areas. For the urban green space, it is recommended that the daily maintenance and management of green spaces be strengthened. For the countryside, it is recommended that the relevant authorities should preserve the integrity of the natural vegetation and ecosystems to enhance the resilience against invasive alien plants. For the farmland, it is recommended that reasonable agricultural cultivation measures be promoted to improve the stability of farmland ecosystems.

## Data Availability

The original contributions presented in the study are included in the article/supplementary material. Further inquiries can be directed to the corresponding author.

## References

[B1] Afonso L. Esler K. J. Gaertner M. Geerts S. (2020). Comparing invasive alien plant community composition between urban, peri-urban and rural areas; the City of Cape Town as a case study. Elsevier. Urban Ecol., 221–236. doi: 10.1016/B978-0-12-820730-7.00013-6

[B2] Beaury E. M. Finn J. T. Corbin J. D. Barr V. Bradley B. A. (2020). Biotic resistance to invasion is ubiquitous across ecosystems of the United States. Ecol. Lett. 23, 476–482. doi: 10.1111/ele.13446 31875651

[B3] Bradley B. A. Blumenthal D. M. Wilcove D. S. Ziska L. H. (2010). Predicting plant invasions in an era of global change. Trends Ecol. Evol. 25, 310–318. doi: 10.1016/j.tree.2009.12.003 20097441

[B4] Cai C. Liu Z. K. Song W. Chen X. Zhang Z. J. Li B.. (2024). Biodiversity increases resistance of grasslands against plant invasions under multiple environmental changes. Nat. Commun. 15, 4506. doi: 10.1038/s41467-024-48876-z 38802365 PMC11130343

[B5] Catford J. A. Jansson R. Nilsson C. (2009). Reducing redundancy in invasion ecology by integrating hypotheses into a single theoretical framework. Divers. Distrib. 15, 22–40. doi: 10.1111/j.1472-4642.2008.00521.x

[B6] Chen P. D. Shen C. C. Tao Z. B. Qin W. C. Huang W. Siemann E. (2024). Deterministic responses of biodiversity to climate change through exotic species invasions. Nat. Plants. 10, 1464–1472. doi: 10.1038/s41477-024-01797-7 39294455 PMC11489087

[B7] Delavaux C. S. Crowther T. W. Zohner C. M. Robmann N. M. Lauber T. van den Hoogen J.. (2023). Native diversity buffers against severity of non-native tree invasions. Nature 622, 773–781. doi: 10.1038/s41586-023-06654-9 PMC10533391 37612513

[B8] Deparis M. Legay N. Isselin-Nondedeu F. Bonthoux S. (2022). Considering urban uses at a fine spatial resolution to understand the distribution of invasive plant species in cities. Landscape Ecol. 37, 1145–1159. doi: 10.1007/s10980-022-01415-x

[B9] Duan T. T. He W. L. Yang J. Q. Lin Z. Y. Lai G. Z. Lu B. C.. (2022). Analysis of the alien invasive plants in Zhanjiang City, Guangdong Province. China. J. Biosaf. 31, 245–251. doi: 10.3969/j.issn.2095-1787.2022.03.008

[B10] Dukes J. S. (2001). Biodiversity and invasibility in grassland microcosms. Oecologia 126, 563–568. doi: 10.1007/s004420000549 28547241

[B11] Erickson A. B. Elton C. S. (1960). The ecology of invasions by animals and plants. J. Wildl. Manage. 24, 231. doi: 10.2307/3796757

[B12] Ernst A. R. Barak R. S. Hipp A. L. Kramer A. T. Marx H. E. Larkin D. J. (2022). The invasion paradox dissolves when using phylogenetic and temporal perspectives. J. Ecol. 110, 443–456. doi: 10.1111/1365-2745.13812

[B13] Faith D. P. (1992). Conservation evaluation and phylogenetic diversity. Biol. Conserv. 61, 1–10. doi: 10.1016/0006-3207(92)91201-3

[B14] Feng Y. Kleunen M. v. (2016). Phylogenetic and functional mechanisms of direct and indirect interactions among alien and native plants. J. Ecol. 104, 1136–1148. doi: 10.1111/1365-2745.12577

[B15] Fridley J. D. Stachowicz J. J. Naeem S. Sax D. F. Seabloom E. W. Smith M. D.. (2007). The invasion paradox: Reconciling pattern and process in species invasions. Ecol 88, 3–17. doi: 10.1890/0012-9658(2007)88[3:Tiprpa]2.0.Co;2 17489447

[B16] Godefroid S. Ricotta C. (2018). Alien plant species do have a clear preference for different land uses within urban environments. Urban Ecosyst. 21, 1189–1198. doi: 10.1007/s11252-018-0792-4

[B17] Guo K. Pyšek P. Chytrý M. Divíšek J. Sychrová M. Lososová Z.. (2024). Stage dependence of Elton’s biotic resistance hypothesis of biological invasions. Nat. Plants. 10, 1484–1492. doi: 10.1038/s41477-024-01790-0 39227727

[B18] Herben T. Mandák B. Bímová K. Münzbergová Z. (2004). Invasibility and species richness of a community: a neutral model and a survey of published data. Ecol 85, 3223–3233. doi: 10.1890/03-0648

[B19] Holzmann K. L. Walls R. L. Wiens J. J. (2023). Accelerating local extinction associated with very recent climate change. Ecol. Lett. 26, 1877–1866. doi: 10.1111/ele.14303 37721806

[B20] Jia J. J. Dai Z. C. Li F. Liu Y. J. (2016). How will global environmental changes affect the growth of alien plants? Front. Plant Sci. 7, 1623. doi: 10.3389/fpls.2016.01623 27847511 PMC5088532

[B21] Karrer S. Teodoro A. D. A. Bustamante M. M. C. Venterink H. O. Edwards P. J. (2020). Species richness both impedes and promotes alien plant invasions in the Brazilian. Sci. Rep. 10, 11365. doi: 10.1038/s41598-020-68412-5 32647221 PMC7347851

[B22] Kraft N. J. B. Comita L. S. Chase J. M. Sanders N. J. Swenson N. G. Crist T. O.. (2011). Disentangling the drivers of β diversity along latitudinal and elevational gradients. Science 333, 1755–1758. doi: 10.1126/science.1208584 21940897

[B23] Levine J. M. (2000). Species diversity and biological invasions: Relating local process to community pattern. Science 288, 852–854. doi: 10.1126/science.288.5467.852 10797006

[B24] Levine J. M. Adler P. B. Yelenik S. G. (2004). A meta-analysis of biotic resistance to exotic plant invasions. Ecol. Lett. 7, 975–989. doi: 10.1111/j.1461-0248.2004.00657.x

[B25] Li J. S. Gao J. X. Zhang X. L. Zheng X. M. (2005). Effects of urbanization on biodiversity: A review. Chin. J. Ecol. 24, 953–957. doi: 10.1111/ele.12522

[B26] Li J. Zhuang C. X. Yang F. F. Lu S. J. Qiu L. S. Zhao J. J. (2024). Characteristics of urban invasive plants and their effects on plant diversity in the built-up area of Shen-zhen, China. Chin. J. Ecol. 43, 2295–2303. doi: 10.13292/j.1000-4890.202408.006

[B27] Li L. X. He R. X. Yang F. Chen J. Cao L. Y. (2023). Effects of urban spatial differences in Haikou on the distribution pattern of invasive alien plants. J. Biosaf. 32, 235–242. doi: 10.3969/j.issn.2095-1787.2023.03.006

[B28] Li S. P. Cadotte M. W. Meiners S. J. Hua Z. S. Shu H. Y. Li J. T.. (2015). The effects of phylogenetic relatedness on invasion success and impact: deconstructing Darwin’s naturalisation conundrum. Ecol. Lett. 18, 1285–1292. doi: 10.1111/ele.12522 26437879

[B29] Liu C. L. Diagne C. A. Angulo E. Banerjee A.-K. Chen Y. Cuthbert R. N.. (2021). Economic costs of biological invasions in Asia. NeoBiota 67, 53–78. doi: 10.3897/neobiota.67.58147

[B30] Liu Y. Speißer B. Knop E. Kleunen M. v. (2022). The Matthew effect: Common species become more common and rare ones become more. Global Change Biol. 28, 3674–3682. doi: 10.1111/gcb.16126 35152520

[B31] Ma J. S. Li H. R. (2018). The checklist of the alien invasive plants in China.

[B32] Ma K. P. Liu Y. M. (1994). Measurement of biotic community diversity I α diversity (Part 2). Biodivers. Sci. 2, 231–239. doi: 10.17520/biods.1994038

[B33] Malecore E. M. Dawson W. Kempel A. Müller G. Kleunen M. v. (2019). Nonlinear effects of phylogenetic distance on early-stage establishment of experimentally introduced plants in grassland communities. J. Ecol. 107, 781–793. doi: 10.1111/1365-2745.13059

[B34] Munemitsu A. Takeshi O. Makihiko I. (2015). The role of roads and urban area in occurrence of an ornamental invasive weed: a case of Rudbeckia laciniata L. Urban Ecosyst. 18, 1021–1030. doi: 10.1007/s11252-015-0466-4

[B35] Mungi N. A. Qureshi Q. Jhala Y. V. (2021). Role of species richness and human impacts in resisting invasive species in tropical forests. J. Ecol. 109, 3308–3321. doi: 10.1111/1365-2745.13751

[B36] Munné-Bosch S. Santos J. A. S. (2024). The dramatic effects of well-intentioned but ill-designed management strategies in plant biological invasions. Nat. Plants. 10, 1148–1152. doi: 10.1038/s41477-024-01747-3 39060424

[B37] Murphy S. M. Vyas D. K. Sher A. A. Grenis K. (2022). Light pollution affects invasive and native plant traits important to plant competition and herbivorous insects. Biol. Invasions. 24, 599–602. doi: 10.5281/zenodo.5676470

[B38] Pearson D. E. Ortega Y. K. Villarreal D. Lekberg Y. Cock M. Eren Ö.. (2018). The fluctuating resource hypothesis explains invasibility, but not exotic advantage following disturbance. Ecol 99, 1296–1305. doi: 10.1002/ecy.2235 29624663

[B39] Potgieter L. J. Gaertner M. Irlich U. M. O’Farrell P. J. Stafford P. J. Stafford L.. (2018). Managing urban plant invasions: a multi-criteria prioritization approach. Environ. Manage. 62, 1168–1185. doi: 10.1007/s00267-018-1088-4 30084019

[B40] Potgieter L. Shrestha N. Cadotte M. W. (2022). Prioritizing terrestrial invasive alien plant species for management in urban ecosystems. J. Appl. Ecol. 59, 872–883. doi: 10.1111/1365-2664.14103

[B41] Qiang H. Ma J. S. (2022). Invasive alien plants in China: An update. Plant Divers. 45, 117–121. doi: 10.1016/j.pld.2022.11.004 36876311 PMC9975470

[B42] Román-Palacios C. Wiens J. J. (2020). Recent responses to climate change reveal the drivers of species extinction and survival. Proc. Natl. Acad. Sci. 117, 4211–4217. doi: 10.1073/pnas.1913007117 PMC7049143 32041877

[B43] Seebens H. Bacher S. Blackburn T. M. Capinha C. Dawson W. Dullinger S.. (2020). Projecting the continental accumulation of alien species through to 2050. Global Change Biol. 27, 970–982. doi: 10.1111/gcb.15333 33000893

[B44] Speißer B. Liu Y. van Kleunen M. (2021). Biomass responses of widely and less-widely naturalized alien plants to artificial light at night. J. Ecol. 109, 1819–1827. doi: 10.1111/1365-2745.1360

[B45] Štajerová K. Šmilauer P. Brůna J. Pyšek P. (2017). Distribution of invasive plants in urban environment is strongly spatially structured. Landscape Ecol. 32, 681–692. doi: 10.1007/s10980-016-0480-9

[B46] Valladares F. Bastias C. C. Godoy O. Granda E. Escudero A. (2015). Species coexistence in a changing world. Front. Plant Sci. 6. doi: 10.3389/fpls.2015.00866 PMC4604266 26528323

[B47] Walsh J. R. Carpenter S. R. Zanden M. J. V. (2016). Invasive species triggers a massive loss of ecosystem services through a trophic cascade. Proc. Natl. Acad. Sci. 113, 4081–4085. doi: 10.1073/pnas.1600366113 27001838 PMC4839401

[B48] Wang X. K. Su Y. B. Ren Y. F. Zhang H. X. Sun X. Ouyang Z. Y. (2020). Urban ecosystem: human and nature compounding. Acta Ecol. Sin. 40, 5093–5102. doi: 10.5846/stxb201901300221

[B49] Wang C. Y. Wu B. D. Jiang K. Zhou J. W. Du D. L. (2019). Canada goldenrod invasion affect taxonomic and functional diversity of plant communities in heterogeneous landscapes in urban ecosystems in East China. Urban For. Urban Green. 38, 145–156. doi: 10.1016/j.ufug.2018.12.006

[B50] Xi L. L. Gou Q. Q. Wang G. H. Song B. (2021). The responses of typical annual herbaceous plants to drought stress in a desert-oasis ecotone. Acta Ecol. Sin. 41, 5425–5434. doi: 10.5846/stxb202006211616

[B51] Yang J. C. Wang G. M. Jiang C. D. Zhao H. T. Zhang Z. D. (2009). Ecological characters and distribution of invasive plants under the influence of urbanization in Beijing, China. Ecol. Environ. 18, 1857–1862. doi: 10.16258/j.cnki.1674-5906(2009)05-1857-06

[B52] Yu W. B. Li S. P. (2020). Modern coexistence theory as a framework for invasion ecology. Biodivers. Sci. 28, 1362–1375. doi: 10.17520/biods.2020243

[B53] Zhang N. (2014). Research on Plant Landscape of Urban Ecological Corridor in Beijing (Beijing Forestry University).

[B54] Zhao Y. F. Zhao C. Y. Zhu J. F. Li F. Yang X. Q. Guo C. D. (2022). Distribution pattern of alien invasive plants in typical parks in Beijing. Acta Ecol. Sin. 42, 3656–3665. doi: 10.5846/stxb202104140971

